# Association of benzodiazepine and Z-drug use with the risk of hospitalisation for fall-related injuries among older people: a nationwide nested case–control study in Taiwan

**DOI:** 10.1186/s12877-017-0530-4

**Published:** 2017-07-11

**Authors:** Nan-Wen Yu, Pei-Jung Chen, Hui-Ju Tsai, Chih-Wan Huang, Yu-Wen Chiu, Wen-Ing Tsay, Jui Hsu, Chia-Ming Chang

**Affiliations:** 1 Department of Psychiatry, Chang Gung Memorial Hospital at Linkou and Chang Gung University, Taoyuan, Taiwan; 2 Division of Rehabilitation & Community Psychiatry, Department of Psychiatry, Chang Gung Memorial Hospital at Taoyuan, Taoyuan, Taiwan; 3 Department of Pediatrics, Feinberg School of Medicine, Northwestern University, Chicago, IL USA; 4 Division of Biostatistics and Bioinformatics, Institute of Population Health Sciences, National Health Research Institutes, Miaoli, Taiwan; 5 Division of Controlled Drugs, Taiwan Food and Drug Administration (TFDA), Ministry of Health and Welfare, Executive Yuan, Taipei, Taiwan

**Keywords:** Benzodiazepine, Z-drugs, Older people, Fall, Hospitalisation

## Abstract

**Background:**

Non-benzodiazepine hypnotics (Z-drugs) are advocated to be safer than benzodiazepines (BZDs). This study comprehensively investigated the association of BZD and Z-drug usage with the risk of hospitalisation for fall-related injuries in older people.

**Methods:**

This study used the Taiwan National Health Insurance Database with a nested matched case-control design. We identified 2238 elderly patients who had been hospitalised for fall-related injuries between 2003 and 2012. They were individually matched (1:4) with a comparison group by age, sex, and index year. Conditional logistic regression was used to determine independent effects of drug characteristics (type of exposure, dosage, half-life, and polypharmacy) on older people.

**Results:**

Older people hospitalisation for fall-related injuries were significantly associated with **c**urrent use of BZDs (adjusted odds ratio [AOR] = 1.32, 95% confidential interval [CI] = 1.17–1.50) and Z-drugs (AOR = 1.24, 95%CI = 1.05–1.48). At all dose levels of BZDs, high dose levels of Z-drugs, long-acting BZD, and short-acting BZD use were all significantly increased the risk of fall-related injuries requiring hospitalisation. Polypharmacy, the use of two or more kinds of BZDs, one kind of BZD plus Z-drugs and two or more kinds of BZDs plus Z-drugs, also significantly increased the risk (AOR = 1.61, 95% CI = 1.38–1.89; AOR = 1.65, 95% CI = 1.08–2.50, and AOR = 1.58, 95% CI = 1.21–2.07).

**Conclusions:**

Different dose levels and half-lives of BZDs, a high dose of Z-drugs, and polypharmacy with BZDs and Z-drugs were associated with an increased risk of fall-related injury requiring hospitalisation in older people. Physicians should balance the risks and benefits when prescribing these drug regimens to older people considering the risk of falls.

## Background

Falls are common in older people and are the second leading cause of accidental or unintentional deaths worldwide [1]. Approximately 28%–35% of people aged 65 years and older fall each year [2], and a study found that fall-related trauma accounts for 5.3% of all hospitalisations in this age range [3]. The risk of falls is multifactorial, and medications are modifiable extrinsic risk factors [4, 5]. Meta-analyses and systematic reviews have reported that some classes of medications, such as benzodiazepines (BZDs), increase the risk of falls in older people [6–8].

BZDs are among the most prescribed psychotropic medications, especially in older people [9]. Because BZDs can be used as sedatives and hypnotics, they can cause problems as dependence and abuse [10] besides the side effects of dizziness, drowsiness, and coordination impairment. Moreover, after controlling the confounding factors, several studies have reported that BZDs independently increase the risk of accidents such as falls [11, 12], hip fractures [13, 14], and motor vehicle accidents [15]. However, data on whether different characteristics (as exposure duration, daily dose, and elimination half-life) lead to different risks are inconsistent. A study reported that the use of long-acting BZDs increases the risk of falls [16], whereas other studies have reported that the use of short-acting BZDs also increases this risk [17, 18]. In addition, another study indicated that dosage contributes more to the risk of falls than elimination half-life [19].

Z-drugs (zolpidem, zopiclone, and zaleplon) are non-BZD hypnotics and are advocated to be safer than BZDs [20]. Thus, the prescription of Z-drugs has been increasing rapidly [21]. A study reported that the annual use of Z-drugs in older people doubled from 2001 to 2010 in Taiwan [22]. However, some studies have demonstrated that Z-drugs also cause problems of abuse and dependence [23], and increase the risk of falls [24] and hip fractures [25].

Polypharmacy is an arising issue in public health and has a crucial role as a risk factor for falls [26]. Studies have found that the simultaneous use of two or more anxiolytics or hypnotics (another kind of polypharmacy) is common in Taiwan [27]. However, whether such polypharmacy with BZDs and Z-drugs increases the risk of fall-related injuries requiring hospitalisation in Taiwan remains unknown.

Since it was found that BZDs and zolpidem (one kind of Z-drugs) were two of the top five reported abused drugs by medical institutions in Taiwan [28] and that their long-term use (defined as 180 prescription days within a year) was not uncommon [29], it is important to understand their potential risk. This study investigated the association of the use of BZDs and Z-drugs with the risk of hospitalisation for fall-related injuries, with a focus on exposure duration, daily dose, elimination half-life, and BZD and Z-drug polypharmacy in older people.

## Methods

### Data sources

This is a nested matched case-control study. We obtained data from the National Health Insurance Research Database (NHIRD) provided by the Ministry of Health and Welfare (MHW) in Taiwan. The Taiwanese government launched the National Health Insurance (NHI) programme in March 1995; the NHI programme covered more than 99% of the total population of Taiwan by the end of 2008 [30]. The NHIRD was developed at the National Health Research Institutes, which linked data from demographic and enrolment records, hospital claims, ambulatory care visits, and pharmacy-dispensing claims from hospitals, outpatient clinics, and community pharmacies. Every person in Taiwan has a unique personal identification number. To secure patients’ confidentiality, the MHW removed all identifiable patient information from the NHIRD. In this study, we used a subset of the NHIRD: Longitudinal Health Insurance Database 2000 (LHID 2000). The LHID 2000 is a data set released by the NHIRD that contains all original claims data for 200 thousand randomly selected beneficiaries in the 2000 Registry of Beneficiaries. The sex and age distributions in the sample were not significantly different from those in the general population.

Our study was approved by the Institutional Review Board of Chang Gung Memorial Hospital (No.103-6020B). No informed consent was required from the subjects because the data were analysed anonymously. We analysed data from 2003 to 2012. There were no BZD and Z-drug prescription policy changes during the study period.

### Study population

#### Definition of cases of hospitalisation for fall-related injuries

Between 2003 and 2012, the Taiwan National Health Insurance System still used the International Classification of Diseases, Ninth Revision, Clinical Modification (ICD-9-CM) codes, and each inpatient has up to 5 discharge codes. The incident cases of hospitalisations for fall-related injuries were defined as subjects aged 65 years and older with discharge diagnosis codes between E-880 and E-888 from 2003 to 2012. The admission date of fall-related hospitalisation was defined as the index date.

#### Definition of the comparison group

The comparison group consisted of subjects randomly selected from the remaining study population who did not have any record of discharge diagnosis of fall-related injury during 2003–2012. These subjects were individually matched with the case subjects according to sex, birth year, and index year at a ratio of 1:4. Each comparison subject was assigned an index date that matched one of the case subjects. If there were less than 4 eligible subjects, they were all included.

**Fig. 1:**
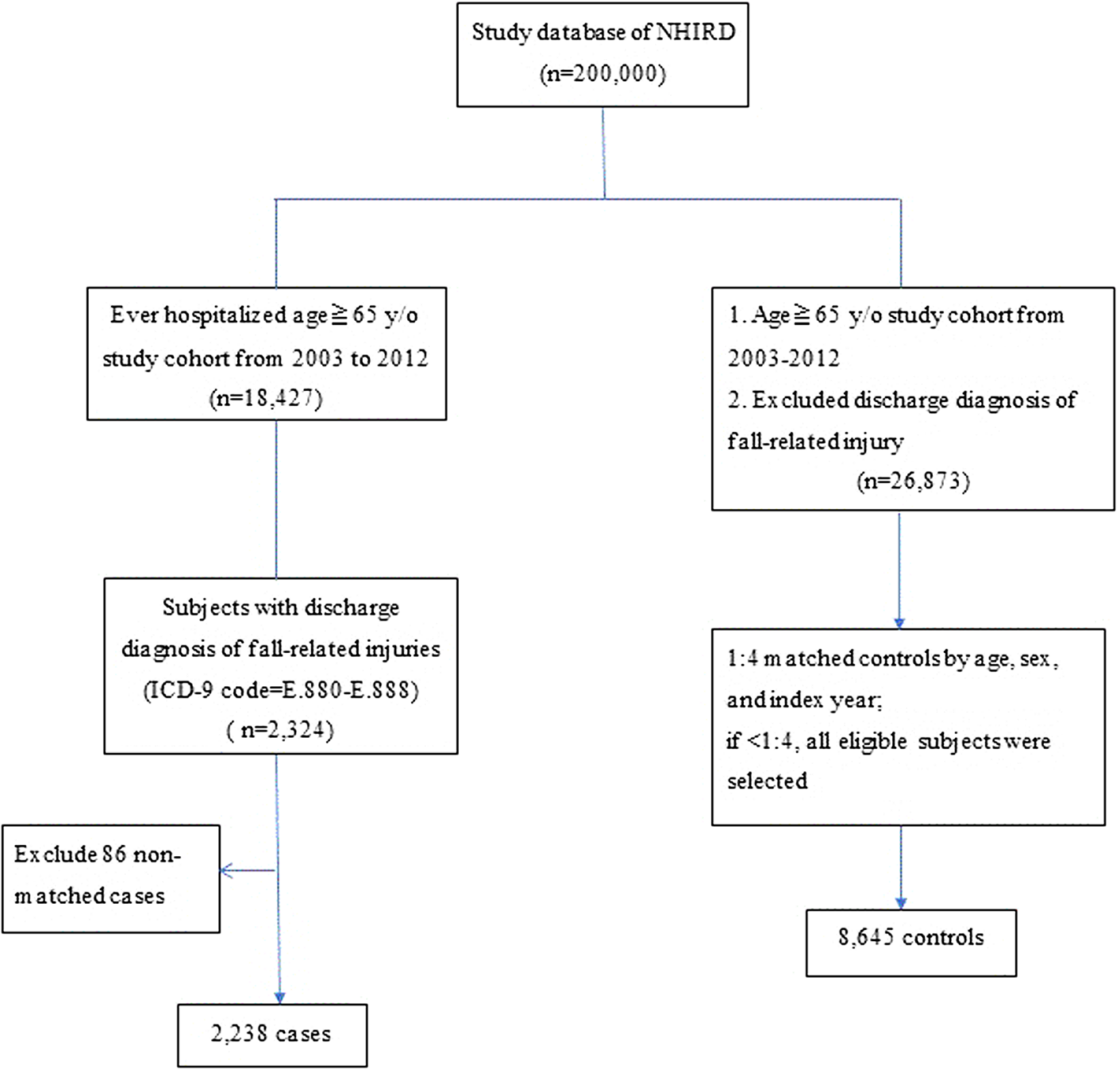
Flow chart of sampling procedure for the study (both case and control group). NHIRD = National Health Insurance Research Database

Initially we identified 2324 eligible case subjects. To avoid missing information, we excluded 86 cases who could not match any controls. As a result, we therefore included 2238 cases and 8645 matched controls (3.86 controls per case) in the subsequent analysis. A detailed flow diagram for identifying the study cohort is showed in Fig. 1.

### Definition of BZD and Z-drug usage

#### Types of BZD and Z-drug exposure

In Taiwan, all the BZDs and Z-drugs are only available with prescriptions from physicians. Following the classification of Ray et al. [31], the types of BZD and Z-drug exposure were classified based on when the most recent fill prescription during the 365 days before the index date and were divided into three groups: 1–30 (current users), 31–90 (indeterminate users), and 91–365 days (former users), respectively. BZD fills on the day of the hospitalisation date were excluded to avoid the problems in the temporality of the exposure-outcome. We defined the group without any BZD or Z-drug prescription within 365 days before the index date as “non-users” group.

#### Dose of BZD and Z-drugs

We calculated the daily dose of BZDs and Z-drugs as a defined daily dose (DDD) of diazepam (1 DDD = 10 mg) for all BZDs and Z-drug prescriptions that overlapped with the index date. We used the WHOCC Index [32] (defined as ATC/DDD) as reference. Based on the distribution of the doses, the subjects were divided into the following three groups: low (<0.3), medium (0.3–0.6), and high (>0.6) DDD [13].

#### Elimination half-life of BZDs and Z-drugs

Diazepam, flurazepam, chlordiazepoxide, and clonazepam were classified as BZDs with long half-life (over 24 h). The other BZDs (alprazolam, bromazepam, brotizolam, clobazam, clorazepate, cloxazolam, estazolam, fludiazepam, flunitrazepam, lorazepam, lormetazepam, medazepam, midazolam, nimetazepam, nitrazepam, nordazepam, oxazepam, oxazolam, prazepam, temazepam, and triazolam) were classified as short half-life agents (less than 24 h).

#### Polypharmacy with BZDs and Z-drugs

According to Wang et al. [27], polypharmacy was defined as exposure to two or more BZDs or any BZDs plus Z-drugs within 30 days before the index date. Since the combination of two or more Z-drugs is very rare, we did not analyze such combinations as polypharmacy.

### Covariates

We used the outpatient claims to identify potential confounders including dementia (ICD-9-CM code 290*), epilepsy (ICD-9-CM 345*), Parkinson’s disease (ICD-9-CM 332*), cerebrovascular disease (ICD-9-CM 430*-438*), diabetes (ICD-9-CM 250*), hypertension (ICD-9-CM 401*-405*), and ischemia heart disease (ICD-9-CM 410*-415*). All prescriptions filled during the 365 days prior to the index date were reviewed to verify the inclusion of antipsychotics, antidepressants, anticonvulsants, thiazide diuretics, and opioids. In addition, we calculated the Charlson comorbidity index scores (CCI) [33] based on the comorbid diagnoses and number of outpatient services received during the 6 months before the index date. The CCI was developed, originally, as a prognostic indicator for patients with a variety of medical conditions and has been commonly used to measure patients’ comorbid conditions.

### Statistical analysis

We first compared the demographic characteristics, comorbidities, exposure to other medications, and health care utilisation between the case and comparison subjects. The unadjusted odds ratio was calculated through bivariate conditional logistic regression. Multivariate analysis was performed using conditional logistic regression to determine the independent effects of BZD and Z-drug characteristics (e.g., type of exposure, dosage, half-life, and polypharmacy) on older people. Multivariate results are reported as adjusted odds ratio (AOR) with 95% confidence interval. Analyses were performed using SAS, version 6.0 (SAS Institute, Inc., Cary, NC).

## Results

The demographic and clinical characteristics of the case and comparison subjects are listed in Table 1. The mean age of the fall patients was 77.7 years, and 60.7% were women. Compared with the control subjects, the case subjects were more likely to have been diagnosed with dementia, Parkinson’s disease, and cerebrovascular disease while controls were more likely to have been diagnosed of hypertension and ischemia heart disease during the 365 days before the index date. In addition, the case subjects were more frequently exposed to antipsychotics, antidepressants, and opioids but less likely to exposed to thiazide diuretics during this study period. Compared with the control subjects, the case subjects had significantly less received outpatient services in the 180 days before the index date.

**Table 1:** Demographic and clinical characteristics of the elderly patients hospitalised for fall injuries

	Case		Controls		Unadjusted OR^a^	95% CI
	*n* = 2238	%	*n* = 8645	%		
Sex					NA	
Female	1359	60.7	5222	60.4		
Male	879	39.3	3423	39.6		
Age, years					NA	
65–74	765	34.2	3046	35.2		
75–84	958	42.8	3800	44.0		
≥ 85	515	23.0	1799	20.8		
Index year					NA	
2003	175	7.8	682	7.9		
2004	214	9.6	836	9.7		
2005	211	9.4	817	9.5		
2006	218	9.7	855	9.9		
2007	257	11.5	1005	11.6		
2008	255	11.4	979	11.3		
2009	248	11.1	959	11.1		
2010	217	9.7	835	9.7		
2011	226	10.1	848	9.8		
2012	217	9.7	829	9.6		
Comorbidities within 365 days before index date						
Dementia	197	8.8	550	6.4	*1.40*	*1.18–1.67*
Epilepsy	25	1.1	94	1.1	1.03	0.66–1.61
Parkinson’s disease	165	7.4	333	3.9	*1.98*	*1.64–2.40*
Cerebrovascular disease	547	24.4	1758	20.3	*1.27*	*1.13–1.42*
Diabetes	629	28.1	2315	26.8	1.09	0.98–1.21
Hypertension	1296	57.9	5661	65.5	*0.73*	*0.66–0.80*
Ischemia Heart Disease	491	21.9	2341	27.1	*0.76*	*0.68–0.85*
Medication exposure within 365 days before index date						
Antipsychotics	328	14.7	1011	11.7	*1.31*	*1.14–1.50*
Antidepressants	386	17.3	1089	12.6	*1.45*	*1.28–1.65*
Anticonvulsants	129	5.8	458	5.3	1.11	0.91–1.36
Thiazide Diuretics	235	10.5	1140	13.2	*0.78*	*0.67–0.90*
Opioids	378	16.9	687	8.0	*2.38*	*2.07–2.73*
	Mean	SD	Mean	SD		
Age (years)	77.7	7.2	77.3	7.0	NA	
Charlson Comorbidity Index score	1.4	1.5	1.5	1.5	0.98	0.95–1.01
Numbers of outpatient services in the 180 days before index date	16.5	13.9	19.6	13.1	*0.98*	*0.98–0.99*

^a^Significant results (*p*<0.05) are in italicize

Table 2 illustrates the relationship between the risk of hospitalisation for fall-related injuries and the types of exposure to BZDs and Z-drugs. The current users of BZDs were significantly associated with a high risk of hospitalisation for fall-related injuries after adjustment for covariates (AOR = 1.32, 95% CI = 1.17–1.50). However, the risk was lower in the indeterminate and former users of BZDs than in the nonusers (AOR = 0.75, 95% CI = 0.62–0.91 for indeterminate users and AOR = 0.74, 95% CI = 0.64–0.86 for former users). Compared with Z-drug non-users, the current users of Z-drugs also showed significantly higher risk of hospitalisation for fall-related injuries after adjusting the covariates (AOR =1.24, 95%CI = 1.05–1.48).

**Table 2:** Unadjusted and adjusted odds ratios of BZD and Z-drug usage in the elderly patients hospitalised for fall injuries according to different type of exposure

	Cases		Controls		Unadjusted		Adjusted^ab^	
	*n* = 2238	%	*n* = 8645	%	OR	95% CI	OR	95% CI
BZDs								
Current users	672	30.0	2144	24.8	1.18	1.06–1.32	*1.32*	*1.17–1.50*
Indeterminate users	160	7.2	879	10.2	0.69	0.57–0.83	*0.75*	*0.62–0.91*
Former users	295	13.2	1519	17.6	0.73	0.63–0.84	*0.74*	*0.64–0.86*
Non-users	1111	49.6	4103	47.5	1		1	
Z-drugs								
Current users	224	10.0	721	8.3	1.23	1.05–1.45	*1.24*	*1.05–1.48*
Indeterminate users	59	2.6	263	3.0	0.89	0.67–1.19	0.87	0.64–1.18
Former users	141	6.3	572	6.6	0.98	0.81–1.18	0.96	0.78–1.17
Non- users	1814	81.1	7089	82.0	1		1	

^a^Adjusted for dementia, Parkinson’s disease, cerebrovascular disease, diabetes, hypertension, ischemia heart disease, antipsychotics, antidepressants, anticonvulsants, thiazide diuretics, opioids, Charlson comorbidity index, and number of outpatient services in the 180 days before the index date

^b^Significant results (*p*<0.05) are in italicize

Table 3 demonstrates the relationship between the risk of hospitalisation for fall-related injuries and the dose levels of BZDs and Z-drugs among the current users. After adjustment for covariates, significantly increased AORs were observed for all dose levels of BZDs users (AOR = 1.75, 95% CI = 1.47–2.08; AOR = 1.54, 95% CI = 1.28–1.85; and AOR = 1.27, 95% CI = 1.08–1.50, respectively, for high, medium, and low dose levels). For the current users of Z-drugs, only the high dose level significantly increased the risk after adjusting the covariates (AOR = 1.37, 95% CI = 1.14–1.64).

**Table 3:** Unadjusted and adjusted odds ratio of BZD and Z-drug usage in the elderly patients hospitalised for fall injuries according to dose levels among current users

	Cases		Controls		Unadjusted		Adjusted^ab^	
	*n* = 2238	%	*n* = 8645	%	OR	95% CI	OR	95% CI
BZDs								
High	237	10.6	651	7.5	1.53	1.31–1.80	*1.75*	*1.47–2.08*
Medium	196	8.8	639	7.4	1.30	1.10–1.54	*1.54*	*1.28–1.85*
Low	239	10.7	854	9.9	1.17	1.00–1.37	*1.27*	*1.08–1.50*
Non-users	1566	70.0	6501	75.2	1		1	
Z-drugs								
High	203	9.1	609	7.0	1.34	1.13–1.58	*1.37*	*1.14–1.64*
Medium	19	0.9	96	1.1	0.78	0.48–1.28	0.77	0.46–1.28
Low	2	0.1	16	0.2	0.50	0.11–2.16	0.46	0.10–2.08
Non-users	2014	90.0	7924	91.7	1		1	

^a^Adjusted for dementia, Parkinson’s disease, cerebrovascular disease, diabetes, hypertension, ischemia heart disease, antipsychotics, antidepressants, anticonvulsants, thiazide diuretics, opioids, Charlson comorbidity index, and number of outpatient services in the 180 days before the index date

^b^Significant results (*p*<0.05) are in italicize

Table 4 shows the relationship between the risk of hospitalisation for fall-related injuries and the characteristics of BZD exposure with respect to the index date, the elimination half-life, and polypharmacy. For the elimination half-life, all three categories, namely long-acting BZDs (AOR = 1.41, 95% CI = 1.16–1.71), short-acting BZDs (AOR = 1.42, 95% CI = 1.20–1.69), and combined long- and short-acting BZDs (AOR = 1.61, 95% CI = 1.37–1.89), significantly increased the risk after adjustment for covariates. Compared with nonusers, treatment with only one kind of BZD and only Z-drugs were associated increased risk of fall-related injury (AOR = 1.40, 95% CI = 1.19–1.65 and AOR = 1.33, 95%CI = 1.04–1.69). Regarding polypharmacy, two or more types of BZD (AOR = 1.61, 95% CI = 1.38–1.89), one kind of BZD+ Z-drugs (AOR = 1.65, 95% CI = 1.08–2.50), and two or more types of BZD plus Z-drugs (AOR = 1.33, 95% CI = 1.04–1.69) significantly increased the risk of hospitalisation for fall-related injuries. The risk of fall-related injury requiring hospitalisations when polypharmacy were all significantly more prominent than monotherapy of only one kind of BZD or only Z-drugs alone.

**Table 4:** Effects of BZD and Z-drug properties, half-life, and polypharmacy on the elderly patients hospitalised for fall injuries

	Cases		Controls		Unadjusted		Adjusted^ab^	
	*n* = 2238	%	*n* = 8645	%	OR	95% CI	OR	95% CI
Elimination half-life of BZD								
Only long-acting BZD	160	7.2	502	5.8	1.35	1.12–1.62	*1.41*	*1.16–1.71*
Only short-acting BZD	213	9.5	692	8.0	1.29	1.10–1.52	*1.42*	*1.20–1.69*
Long- + short- acting BZD	299	13.4	950	11.0	1.33	1.15–1.53	*1.61*	*1.37–1.89*
Non-users	1566	70.0	6501	75.2	1		1	
Polypharmacy of BZD and Z-drugs								
Only one kind of BZD	238	10.6	781	9.0	1.30	1.11–1.52	*1.40*	*1.19–1.65*
Two or more kinds of BZD	309	13.8	979	11.3	1.35	1.17–1.55	*1.61*	*1.38–1.89*
Only Z-drugs	99	4.4	337	3.9	1.26	1.00–1.59	*1.33*	*1.04–1.69*
One kind of BZD + Z-drugs	34	1.5	89	1.0	1.61	1.08–2.40	*1.65*	*1.08–2.50*
Two or more kinds of BZD + Z-drugs	91	4.1	295	3.4	1.33	1.04–1.70	*1.58*	*1.21–2.07*
Non-users	1467	65.6	6164	71.3	1		1	

^a^Adjusted for dementia, Parkinson’s disease, cerebrovascular disease, diabetes, hypertension, ischemia heart disease, antipsychotics, antidepressants, anticonvulsants, thiazide diuretics, opioids, Charlson comorbidity index, and number of outpatient services in the 180 days before the index date

^b^Significant results (*p*<0.05) are in italicize

## Discussion

In this population-based study, we observed that the current use of BZDs was associated with an increased risk of fall-related injuries requiring hospitalisation in older people, irrespective of dose levels (low, medium, or high) and elimination half-life (long-acting or short-acting). By contrast, the current use of Z-drugs, in particular at high dose levels increased this risk. With respect to polypharmacy, the use of more than one type of BZD significantly increased the risk, which reached the highest prominence when two or more types of BZD plus Z-drugs were used.

Our study results revealed that even low doses (<0.3 DDD) of BZDs were associated with an increased risk of fall-related injuries requiring hospitalisation among older people, and this risk correlated with an increase in the dose. A study [19] reported that a BZD daily dose higher than 0.75 DDD increased the risk of falls leading to femur fractures among subjects aged 55 years and older. The results of our previous study [13] and another study [14] demonstrated that a BZD daily dose higher than 0.3 DDD increased the risk of hip fractures in older people. These studies suggest that physicians should avoid prescribing BZDs to older people considering the high risk of falls even on low dose.

The 2002 Beers criteria [34] suggested that physicians should avoid prescribing long-acting BZDs to adults aged 65 years and older because they are potentially inappropriate medications. However, the 2012 and 2015 Beers criteria [35, 36] further suggested that the prescription of any BZD to older people is potentially inappropriate. Our data are consistent with those of previous studies [13, 14, 18] and support the notion that short-acting BZDs are not safer than long-acting BZDs in older people.

Previous studies have reported that zolpidem increased the risk of falls [24] or fractures [25] in older people, our results also indicated that the current use of Z-drugs, in particular at high dose levels (>0.6 DDD) increased the risk of hospitalisation for fall-related injuries in older people.

In this study, we observed that the concomitant use of two or more types of BZD or any BZDs plus Z-drugs were significantly associated with the risk of hospitalisation for fall-related injuries among older people. Such definition of polypharmacy may differ from other studies [26, 37], which define it as the combined use of 5 or more drugs daily. Because such anxiolytic-hypnotic polypharmacy is common and has increased in Taiwan [27], more attention should be focused on the potential risk of falls in older people.

Two unexpected findings were noted in this study. First, the cases were less numbers of outpatient services in the 180 days before the index date. It is possible due to a bias in the selection of controls (e.g., some controls may have severe physical comorbidities which make them totally bed ridden and less prone to falls). Second, compared with BZDs non-users, indeterminate users and former users seemed to have lower risk of fall-related injuries than non-users. Such results may be due to some uncontrolled confounding factor. However, the indeterminate and former users may have protected effects which suggest older people who stop BZDs may reduce the risk of fall-related hospitalisations. Several limitations should be considered before interpreting our results. First, since the patients with less severe fall-related injuries may not have been recorded in the NHIRD, we used E-code to define the outcome. However, the E-code may not routinely be recorded by physicians in Taiwan and may have poor sensitivity. Such definition may underestimate or overestimate the fall risk of BZDs / Z-drugs to the elderly. Second, we defined BZDs and Z-drugs exposure within 1–30 days before the index date as “current use”, which followed the classification of Ray et al. [31] in their earlier paper. However, they proposed the potentially serious misclassification in the study of the “acute effects” of BZDs and other drugs used intermittently upon studies of related injuries and suggested to track exposure on a day-by-day basis in future studies [38]. Third, although we used CCI and healthcare utilizations to control the comorbidities, some additional residual confounding factors should be considered such as vision problems, body mass index, physical activity, smoking, and alcohol use which may not be available from our data. Additionally, the functional abilities (gait speed, difficulties with activities of daily living) would be also important but not be available with administrative data. Forth, because the NHIRD only provided prescription records of drugs, we are unable to assert the exactness of each subject’s medicating status, as noncompliance or “on a need basis” are also possible. Last, confounding by indication is another major bias in the observatory pharmacoepidemiological studies and may underestimate or overestimate the risk between BZDs/Z-drugs and fall in elderly people.

Despite several limitations listed above, our study has several strengths. The sample size used (NHIRD) was large, as it consists of a nationwide registry for medical claims data in Taiwan. In addition, ours is one of the few studies simultaneously examining the specific characteristics of BZD and Z-drug use among older people in a single study, with a focus on the type of exposure, daily dose, elimination half-life, and polypharmacy. Additionally, this is the first study to investigate the effects of polypharmacy with BZDs and Z-drugs on the risk of hospitalisation for fall-related injuries among older people. Consideration that Z-drugs are increasingly used as a common treatment choice for insomnia and the combined use of BZDs and Z-drugs is increasing in older people, the focus of this study is relevant for clinical decisions.

## Conclusion

In conclusion, this study shows that different dose levels and half-lives of BZDs, a high dose of Z-drugs, and polypharmacy with BZDs and Z-drugs were associated with an increased risk of fall-related injury requiring hospitalisation in older people. Physicians should balance the risks and benefits when prescribing these drug regimens to older people considering the risk of falls.

## Abbreviations

AOR: adjusted odds ration
ATC: Anatomical Therapeutic Chemical
BZD: benzodiazepine
CCI: Charlson comorbidity index
CI: confidential interval
DDD: defined daily dose
ICD-9-CM: International Classification of Disease, Ninth Revision, Clinical Modification
LHID: Longitudinal Health Insurance Database
MHW: Ministry of Health and Welfare
NHI: National Health Insurance
NHIRD: National Health Insurance Research Database
WHOCC: World Health Organization Collaborating Centre
